# Factors Associated with Underweight among Under-Five Children in Eastern Nepal: Community-Based Cross-sectional Study

**DOI:** 10.3389/fpubh.2017.00350

**Published:** 2017-12-22

**Authors:** Deepak Adhikari, Resham Bahadur Khatri, Yuba Raj Paudel, Amod Kumar Poudyal

**Affiliations:** ^1^Ministry of Health and Population, Kathmandu, Nepal; ^2^Faculty of Medicine, School of Public Health, The University of Queensland, Brisbane, QLD, Australia; ^3^Nepal Health Sector Support Program, Ministry of Health and Population, Kathmandu, Nepal; ^4^Department of Community Medicine and Public Health, Institute of Medicine, Tribhuvan University, Kathmandu, Nepal

**Keywords:** environmental factors, maternal factors, Nepal, underweight, under-five children

## Abstract

**Background:**

Undernutrition is a leading cause of morbidity and mortality in children in developing countries including Nepal. This study aimed to identify sociodemographic, environmental, and maternal and child health (MCH) factors associated with objectively assessed underweight among children aged under 5 years in Ilam district of eastern Nepal.

**Methods:**

A community-based cross-sectional study of 300 mothers of children under 5 years was conducted using interviewer-administered questionnaires from July to August 2012. The sample was derived by randomly selecting three village development committees (VDCs), then three wards from each of these three VDCs were selected making a total sample of nine wards. Finally, individuals were selected from the nine wards using systematic random sampling. Chi-square tests were used to identify factors associated with childhood underweight. Logistic regression analyses were conducted to determine odds ratios for the factors associated with underweight.

**Results:**

The prevalence of underweight was 37% [95% confidence interval (CI): 33–43%]. Children who were more than 24 months of age were more likely to be underweight (adjusted odds ratio (aOR) = 2.72; 95% CI: 1.57, 4.70) than children aged less than 24 months. Children of families who consumed water without treatment had higher odds of being underweight (aOR = 2.48; 95% CI: 1.28, 4.78) than those who used water after boiling. Children whose mother perceived their size at birth as normal were more likely to be normal weight (aOR = 0.40; 95% CI: 0.16, 0.99) compared to a smaller size at birth. Children whose growth was monitored had a low chance of being underweight (aOR = 0.35, 95% CI: 0.15, 0.97).

**Conclusion:**

Nearly two-fifth of under-five children were found to be underweight. The age of children, drinking water purification practices, growth monitoring, and mother’s perception of size at birth were significantly associated with childhood underweight. These findings suggest that interventions focusing on access to child growth monitoring, and water and sanitation practices may reduce the childhood underweight.

## Introduction

Maternal and child undernutrition causes more than 10% of the total global burden of disease and more than one-third of child deaths (1, 2). The poor nutritional status of children and mothers has long-term health consequences, and the ramifications extend to inter-generational low productivity and perpetuation of poverty (3–6). Despite growing emphasis on promotion of maternal and child nutrition through various policies, plans, and programs (5, 7), Nepal has not made significant progress in the reduction of maternal and child undernutrition as expected (8, 9). For example, stunting in under-five children declined from 57% in 2001 to 36% in 2016, and underweight in under-five children declined from 43% in 2001 to 27% in 2016 (9). Global literature has revealed that 45% of under-five deaths are contributed by undernutrition as malnourished children are highly susceptible to childhood diseases (10). Therefore, reduction of underweight can be an important step toward reducing childhood morbidity and mortality in Nepal.

Multiple factors affect childhood nutritional and development status, including household socioeconomic position, child feeding practices, environmental factors, access to and use of health services, and household level hygiene practices (2). Childhood illnesses such as diarrhea and acute respiratory infections, which are associated with poor hygiene and access to sanitation, are common causes of under-nutrition in developing countries including Nepal (11–14). It has been suggested that nutritional interventions and environmental sanitation strategies such as access to clean water, waste disposal, and use of clean household fuels can reduce childhood underweight and reduce child mortality by 14–31% in developing countries in South Asia and Africa (15).

While there has been some research on the effect of feeding practices and socioeconomic conditions (14, 16–18) on childhood undernutrition in Nepal, there is little empirical research on how socioeconomic and environmental conditions may influence childhood underweight. Furthermore, understanding these relationships in rural settings may offer evidence for designing contextual promotion activities. Thus, this paper investigates the association between social, environmental, and maternal and child health (MCH) factors and low weight for age (weight for age *Z* score <−2 SD) among children under 5 years old from a rural district of eastern Nepal.

## Materials and Methods

### Study Setting

This cross-sectional study was conducted in Ilam district of eastern Nepal from July to August 2012. Ilam district is one of 75 districts and lies to the east of the country. People from indigenous ethnicities (*Rai, Limbu, Rajbansi*) represent more than two-third of the population (70%) in the district. About 72% females are literate in Ilam, which is one of the highest ranked districts of Nepal regarding the United Nation’s Human Development Index (19, 20). The agricultural activities especially tea and livestock farming are the major sources of income of people living in Illam district. It has three electoral constituencies and 48 village development committees (VDCs)—the latter being the smallest administrative units in Nepal (20). Each VDC is further divided into nine wards for service delivery and development purposes.

### Sampling and Sample Size

Three VDCs, namely Chulachuli, Godak, and Chamaita, were randomly selected from the list of 48 VDCs, representing each of the three electoral constituencies. Three wards from each participating VDC were then randomly selected resulting in a total sample of 9 wards. Secretaries (administrative in-charge of VDC) and health post in-charges from the selected VDCs were briefed on the research, and they provided their consent to be involved. In Nepal, vitamin A and polio immunization services are provided routinely as well as biannually as national campaigns. The coverage of those services is almost universal among under-five children. Therefore, a sampling frame of children aged under 5 years was prepared from the selected wards by using the vitamin A and polio immunization register of female community health volunteers (FCHVs) from each ward. This resulted in a sampling frame of 910 households from nine wards of three VDCs. The sample size of 300 was determined based upon an alpha of 0.05, 29% prevalence of underweight as per Nepal Demographic and Health Survey (NDHS) 2011, and non-response rate of 10% using the Epi-Info Stat Calc version 7. The number of participating households per ward ranged from 19 to 54. Systematic sampling (every fourth household) was used for interview and anthropometric measurement.

Mothers of children aged under 5 years were selected for an interview, and the research team undertook anthropometric measurement of the children. If the mother of a child aged under 5 years was not available in the selected households, the adjoining household was recruited, and second visits were undertaken if such household did not have an under-five child. Only five such households which have no under-five children were found during the data collection. If there was more than one eligible child in the same family, the elder child was selected for weight-for-age measurement. If it was not possible to interview the mother in the first visit, a second visit was made to interview the mother. All mothers approached responded to the interviews.

### Study Variables and Measurement

The outcome variable was objectively assessed underweight of children aged under 5 years. Underweight was categorized using the World Health Organization growth chart for children and was defined as being <−2 SD from the median reference weight for age (21). Explanatory variables such as sociodemographic factors were mainly self-reported by the child’s mother during the interview. However, environmental factors such as the availability of latrines and cooking fuel were observed by interviewers on the day of the interview. Behavior and practice-related variables were assessed through 24-h recalls of selected mothers. The major explanatory and outcome variables are shown in Table 1.

**Table 1:** Definition of outcome and explanatory variables.

SN	Variables	Categories of variables
**Outcome variable**		

1	Nutrition status of children	Underweight; normal

**Explanatory variables**		

**Sociodemographic factors**		
2	Ethnicity	Disadvantaged (Dalit and Muslim); relatively advantaged (non-Dalit and advantaged Hill castes Janajatis and upper caste group)
3	Sex of child	Male; female
4	Child age	0–23 months; 24–59 months
5	Birth interval of children	None (first child); below 36 months; 36 months or above
6	Maternal age	(≥20 years; less than 20 years
7	Household size	<5 members; above five members
8	Occupation	Agriculture; business; labor work
10	Maternal education	Illiterate; primary education (1–8 grade) and secondary education (9–12 grade)
11	Area of land owned	Landless; up to 0.5 Hectors; more than 0.5 Hector
12	Food sufficiency around the year	Yes; no

**Environmental factors**		
13	Source of drinking water	Improved (tap, covered well); not improved (open well, river)
14	Water purified in last 24 h	Boiling; filtration; and no treatment
15	Types of toilet	No latrine, flush latrine; pit latrine
16	Types of cooking fuel	Solid fuel liquid fuel
17	Soap used in previous 24 h before interview	Yes; no
18	Number of sleeping rooms	<2 rooms; ≥2 rooms

**Maternal and child health factors**		
19	Perceived size at birth	Small; normal
20	Weighted at birth	Yes; no
21	Antenatal check up	No, 1–3 ANC visits, 4 or more visits
22	Place of delivery	Health facility; home
23	Postnatal checkup	Yes; no
24	Growth monitoring in previous six months before interview	Yes; no
25	Child diarrhea in the last 30 days	Yes; no

### Data Collection Tools and Techniques

Three field assistants from each VDC were recruited to assist with data collection. The first author provided a 2-day orientation to the field assistants on interviewing, data recording, and anthropometric measurement (weight of the children). The interviewer-administered questionnaire was conducted with the mothers of children aged under 5 years. The questionnaire was based on relevant literature and the previous surveys such as the NDHS (8, 18, 22). The questionnaire was pretested with ten mothers in the adjoining VDC; some minor changes were made in the language according to the local context. Each field assistant checked data for completion and consistencies after each interview, and the researcher later checked the completed questionnaires at the end of the day.

The weight of a child was measured using a standardized calibrated Salter 235-6S Hanging scale, which can measure up to 0.1 kg. The child’s weight was taken without shoes and with light clothes. The weight of children aged less than 6 months was taken by infant weighing scale (infant spring scale) that is used in community-based newborn care program in Nepal (23).

### Data Entry and Analysis

Data were entered in Epi-data (The EpiData Association, Odense, Denmark) (24), and analyses were conducted using Statistical Package for Social Sciences (SPSS) version 19 (Armonk, NY, USA: IBM Corp.) (25). Descriptive analyses were conducted including proportions of childhood underweight.

To determine the factors associated with underweight, a two-step modeling approach was undertaken. First, univariate logistic regression analyses of each explanatory variable and underweight were conducted to identify the associations. Second, those variables with *p*-values < 0.1 were included in a multivariate logistic regression analysis to determine the effect adjusted for each of the other potential explanatory variables. Multicollinearity was assessed through analysis of the correlation coefficients in the correlation matrix. A *p*-value of <0.05 denoted statistical significance in the final model.

### Ethics Approval and Consent to Participate

Ethical approval from the Institutional Review Board (IRB) of the Institute of Medicine, Tribhuvan University, was obtained for this study. Before collection of the data, permission from the District Health Office, Ilam was obtained. Health workers from each VDC as well as FCHVs from each ward were briefed on the purpose of the study. This study used verbal informed consent with prior approval from the IRB to include the illiterate mothers. Further, it was explained that mothers could terminate the interview at any time. Before starting the interview, the IRB-approved verbal informed consent form was read by the research assistant, and mothers were asked to agree to participate in an interview and anthropometric measurement of their children. Those who agreed had their names, and the name of their child, recorded in the respondents’ summary sheet. After completing this procedure, the interview commenced, and the weight of the child was taken.

## Results

The prevalence of underweight in the study population was 37% [95% confidence interval (CI): 33–43%] (table is not shown).

### Sociodemographic Characteristics of the Sample

The sociodemographic features of the study population are presented in Table 2. Nearly, three quarters of the children (71%) were from the disadvantaged ethnic groups. Sixty percent of the participating children were male, and just over half (51%) were less than 23 months of age. Forty-nine percent of children were the first child. Among the 152 children who had a sibling, 28% had children birth interval of more than 36 months. More than half (54%) of the mothers had given birth to their first child before they were 20 years old. Two-third (66%) of the study population had ≤5 household members, and 21% of responding mothers were illiterate.

**Table 2:** Sociodemographic factors and underweight status of children aged under five years in Ilam district Nepal.

Sociodemographic factors	Categories	Total number (%)^a^	Underweight children (%)^b^	*p*-Value^#^
Ethnicity of mothers	Disadvantaged group	212 (70.6)	81 (38.2)	0.62
	Relatively advantaged group	88 (29.4)	31 (35.2)	
Sex of the child	Male	181 (60.3)	73 (40.3)	0.18
	Female	119 (39.7)	39 (32.8)	
Age group of children	0–23 months	154 (51.3)	44 (28.6)	<0.001*
	24–59 months	146 (48.7)	68(46.6)	
Birth interval of index child	None (first child)	148 (49.3)	53 (35.8)	0.38
	12–36 months	69 (23.0)	23 (33.3)	
	≥36 months	83 (27.7)	36 (43.4)	
Mother’s age at first child birth	≥20 years	138 (46.0)	50 (36.2)	0.71
	<20 years	162 (54.0)	62 (38.3)	
Household size	≤5	197 (65.7)	76 (38.6)	0.61
	>5	103 (34.3)	36 (35.0)	
Main maternal occupation	Agriculture	247 (82.3)	89 (36)	0.31
	Business	23 (7.7)	8 (34.8)	
	Labor	30 (10)	15 (50)	
Maternal education	Illiterate	62 (20.7)	31 (50.0)	<0.001*
	Primary education	41 (13.7)	20 (48.8)	
	Secondary education	197 (65.6)	61 (31.0)	
Area of land owned	Landless	30 (10.0)	19 (63.3)	<0.007*
	Up to 0.5 ha	167 (55.7)	61 (36.5)	
	≥0.5 ha	103 (34.3)	32 (31.1)	
Food sufficiency around the year	No	115 (39.0)	38 (49.3)	0.22
	Yes	185 (61.0)	74 (40.0)	

*^a^Column percentage*.

*^b^Row percentage*.

*^#^Chi-square test of association*.

*p < 0.05.

In univariate analyses, three sociodemographic variables; the age of the child, mother’s education, and area of land owned were associated with the child being underweight.

### Environmental and MCH Factors

Household environmental condition and MCH factors are presented in Table 3. Ninety-one percent of the households had improved sources of water, and 42% of families had consumed water without household-level treatment within the 24 h preceding the survey. About 80% of the households used solid fuel for cooking. Ninety-six percent of households had a latrine. The use of soap during hand washing was practised in 94% of the surveyed households.

**Table 3:** Environmental, maternal and child health (MCH) factors and underweight status of children aged under 5 years in Ilam district Nepal.

Environmental factors	Categories	Total number (%)^a^	Underweight children (%)^b^	*p*-Value^#^
Main source of drinking water	Improved source	273 (91.0)	100 (36.6)	0.41
	Not improved source	27 (9.0)	12 (57.1)	
Water purified in past 24 h	Boiling	143 (47.7)	49 (34.3)	0.07*
	Filtration	30 (10.0)	6 (20.0)	
	No treatment	127 (42.3)	57 (44.9)	
Types of toilet	Flush latrine	146 (48.7)	41 (28.1)	<0.001*
	Pit latrine	141 (47.0)	63 (44.7)	
	No latrine	13 (4.3)	8 (61.5)	
Type of cooking fuel used	Solid fuel	238 (79.3)	95 (39.9)	0.07*
	Liquid fuel	62 (20.7)	17 (27.4)	
Hand washed with soap and water in previous 24 h	No	19 (6.3)	11 (58.0)	0.06*
	Yes	281 (93.7)	101 (35.0)	
Number of sleeping rooms	<2 rooms	59 (19.7)	34 (57.6)	<0.00*
	≥2 rooms	241 (80.3)	78 (32.4)	
Maternal and child health factors				
Perceived size at birth	Small	30 (10)	18 (60.0)	<0.001*
	Normal	270 (90)	94 (34.8)	
Weighed at birth	No	132 (44)	59 (44.7)	0.02*
	Yes	168 (56)	53 (31.5)	
Antenatal care	No	19 (6.3)	13 (68.4)	0.07
	1–3 visits	90 (30.0)	41 (45.6)	
	4 more visits	191 (63.7)	58 (30.3)	
Place of delivery	Home	135 (45)	60 (44.6)	0.022*
	Health facility	165 (55)	52 (31.5)	
Postnatal care	No	121 (40.3)	54 (44.6)	0.032*
	Yes	179 (59.7)	58 (32.4)	
Child growth monitoring in past six months	No	35 (11.7)	21 (60.0)	<0.01*
	Yes	265 (88.3)	91 (34.3)	
Diarrhea in past 30 days before interview	No	224 (75.0)	81 (36)	0.49
	Yes	76 (25.0)	31(41)	

*^a^Column percentage*.

*^b^Row percentage*.

*^#^Chi-square test of association*.

*p < 0.05.

Ninety-four percent of mothers had received antenatal care services during their last pregnancy. More than half (55%) of the responding mothers had given birth at a health facility. During the 6 months before the interviews, nine out of 10 children had their weight monitored. About a quarter of the children had experienced diarrhea in the 30 days prior to the survey.

Five environmental factors (household water purification practices, types of the latrine, types of cooking fuel, handwashing with soap and water in the previous 24 h, and the number of sleeping rooms in the house) were associated with underweight among children aged under 5 years (Tables 2 and 3). Similarly, six MCH-related factors were associated with underweight, including place of childbirth (or delivery), antenatal care visits, post-natal care visits, perceived size at birth, weighing at birth, and growth monitoring practices in the last 6 months (Tables 2 and 3).

The multivariate model is shown in Table 4. After checking for multicollinearity, it was found that weighing at birth and place of childbirth were highly correlated (correlation coefficient = 0.92), therefore, weighing at birth was not included in the multivariate model. Out of 13 explanatory variables, only four were significantly associated with childhood underweight. Age group of the child was associated with underweight, with children aged more than 24 months more likely to be underweight (aOR = 2.72; 95% CI: 1.57, 4.70; *p* < 0.00) than children aged 0–23 months. Children of families who consumed water without treatment had higher odds of being underweight (aOR = 2.48; 95% CI: 1.28, 4.78; *p* = 0.005) than those who consumed boiled water. Mother’s perception of the size of her child at birth was associated with underweight. Children perceived to be of normal size at birth were less likely to be underweight compared to the children perceived to be small at birth (aOR = 0.40; 95% CI: 0.16, 0.99; *p* = 0.048). Likewise, children who were growth monitored had lower odds of being underweight than those who were not growth monitored (aOR = 0.35, 95% CI: 0.15, 0.97; *p* = 0.024).

**Table 4:** Factors associated with underweight among children aged under 5 years in Ilam district Nepal.

Characteristics	Unadjusted odds ratio (OR) [95% confidence interval (CI)]	*p*-Value	Adjusted OR (95% CI)	*p*-Value
Age group of children in months (ref. 0–23 months)		<0.001		<0.00*
24–59	2.18 (1.35, 3.51)		2.72 (1.57, 4.70)	
Maternal education (ref. illiterate)		<0.001		0.19
Primary education	0.69 (0.37, 1.28)		0.82 (0.40, 1.67)	
Secondary education	0.36 (0.19, 0.69)		0.59 (0.26, 1.33)	
Area of land owned (ref. landless)		<0.007		0.18
Up to 0.5 ha	0.33 (0.17, 0.75)		0.38 (0.15, 1.008)	
≥0.5 ha	0.26 (0.11, 0.61)		0.39 (0.15, 1.08	
Water purified in past 24 h (ref. boiling)		<0.07		0.005*
Filtration	0.48 (0.18, 1.25)		0.93 (0.32, 2.69)	
No treatment	1.56 (0.95, 2.55)		2.48 (1.28, 4.78)	
Types of toilet (ref. no latrine)		<0.001		0.20
Pit latrine	0.50 (0.16, 1.61)		1.55 (0.34, 6.98)	
Flush latrine	0.24 (0.07, 0.79)		0.97 (0.20, 4.55)	
Type of cooking fuel used (ref. liquid fuel)		0.07		0.446
Solid fuel	1.75 (0.95, 3.25)		1.36 (0.61, 3.05)	
Soap used during hand washing in past 24 h (ref. no)		0.06		0.237
Yes	0.40 (0.16, 1.04)		0.48 (0.14, 1.61)	
Number of sleeping room (ref. <2 rooms)		<0.000		0.59
≥2 rooms	0.35 (0.19, 0.63)		1.17 (0.64, 2.15)	
Perceived size at birth (ref. small)		<0.001		0.048*
Normal	0.35 (0.16, 0.77)		0.40 (0.16, 0.99)	
Antenatal care (ref. no)		<0.000		0.09
1–3 times	0.38 (0.13, 1.10)		0.54 (0.14, 2.03)	
4 or more	0.20 (0.07, 0.55)		0.36 (0.09, 1.39)	
Place of delivery (ref. home)		0.022		0.41
Health facility	0.57 (0.35, 0.92)		1.40 (0.61, 3.19)	
Postnatal care (ref. no)				
Yes	0.59 (0.37, 0.95)	0.032	0.89 (0.40, 1.99)	0.79
Child growth monitored in past 6 months (ref. no)		0.004		0.024*
Yes	0.35 (0.17, 0.72)		0.35 (0.15, 0.97)	

*p < 0.05, significance in multivariate analysis.

*Ref., reference*.

## Discussion

The prevalence of underweight (weight for age < −2 SD) was found to be 37% (95% CI: 32–43) in the current study. This is higher than the national average (27%) and that of the eastern region of Nepal (24%) (9). More than two-thirds of our sample were from the disadvantaged ethnic communities. Those communities usually have low socioeconomic status, lower land ownership, high rates of childbirth at home, and childhood illnesses, which might be accounted for the prevalence of underweight of children aged under 5 years (26, 27). Pooled figures of three consecutive NDHS data showed higher odds of early initiation of breastfeeding among mothers with primary education (aOR: 1.24) and secondary or higher education (aOR: 1.63) (27). In our study, about one-third of mothers were either illiterate or only had primary level education, and nearly half of their children were underweight. Lower maternal education has been shown to be associated with late initiation of breastfeeding after birth (27) and poor complementary feeding practices of children aged under 2 years, consequently affecting childhood nutritional status (28, 29). Furthermore, mothers who belong to the disadvantaged ethnic groups face food insufficiency around the year and are more likely to engage in labor works for subsistence in Nepal (30). Because of various household chores, mothers from such ethnic groups have less time for child feeding, child caring, and health-seeking during an illness in comparison to the advantaged, educated and wealthy mothers. This consequently affects dietary frequency and diversity in infants and young children (26, 29). Context-specific health information education and communication (IEC)/behavior change communication (BCC) materials such as radio jingle, posters, pamphlets, and other pictorials can be designed in the local languages and delivered through multiple channels so that message can be reached out to disadvantaged communities.

In the current study, children aged 24–59 months were more likely to be underweight than children aged 0–23 months. A survey carried out in Lao PDR found that children in higher age groups had higher odds of underweight, which is consistent with the current study finding (31, 32). Poor socioeconomic status and housing conditions in our study may have contributed to the high prevalence of underweight among children aged 24 months or above. In the rural Nepali contexts, mothers usually give less attention to their children when they reach 2 years of age. At that point, elder siblings or other members of the family take over as their caregivers, which might lead to poor feeding and hygiene practices. As a result, such children are more susceptible to childhood illness, ultimately become underweight (33). Furthermore, nearly one-third of children between the age of 6 and 9 months (30%) do not receive complementary foods in addition to breastfeeding (12). Such practices are more prevalent among poor and marginalized families, which might also be contributing to children being underweight. This finding suggests that later age within under 5 years’ age carry a higher risk of underweight. They need more attention, especially in the frequent feeding of diversified foodstuff in adequate quantity during pre-school period of the children, and the practice should be promoted.

In this study, children from families who consumed water without any treatment, such as boiling, or filtration were found to be more underweight. Poor hygiene and sanitation practices are primarily responsible for childhood illnesses worldwide (34). In this study, about 25% of children had suffered from diarrhea 1 month prior to the data collection. The higher number of diarrheal cases is reported during the summer season in the district where our study was carried out. Moreover, mothers must do their household chores, in addition to being involved in agricultural activities and labor work for subsistence. This also contributes to poor child caring and such children are more prone to undernutrition (35). Moreover, poor child caring practices increases susceptibility to childhood illnesses such as diarrhea and worm infestations which also leads to underweight (36).

In the current study, the likelihood of being underweight was higher among those children whose mother perceived smaller size at birth than average—a finding consistent with a study conducted in 2014 in Nepal (37). Mothers’ nutritional status determines the nutrition of their children at birth (38). In Nepal, about half of the pregnant women are anemic or have low body mass index (12). A randomized controlled trial conducted in Nepal reported that the supplementation of multiple micronutrients during pregnancy increased the birth weight and reduced low birth by 14% (39). However, there is a widespread cultural belief that if a pregnant woman consumes a lot of nutritious food during her pregnancy, the baby growing in her womb will become very big in size which will then make childbirth more painful and difficult (40). Such belief subjects mothers to nutritional deficiency during pregnancy which might result in intrauterine growth retardation, preterm births, and/or children being born with small size at births. If parents perceive that their newborn is smaller than average or is born with low birth weight, such babies need additional postnatal care and frequent feedings because such children face higher chance of becoming ill (39, 41). So, maternal nutrition education and counseling session during antenatal care visits should be promoted focusing on undernourished pregnant women. Parents of low birth weight, preterm, and small babies also need to be made aware of the future implications. The need for adequate attention and nutritional support, caring, and health seeking of such children should also be highlighted.

The children who were not growth-monitored in the last 6 months preceding the survey were over two times more likely to be underweight than those who were monitored. Studies have shown that regular growth monitoring is associated with better feeding practices of children (18, 42). A review of growth monitoring and promotion impact conducted in 2008 reported that growth monitoring could provide an entry point for preventive and curative health care and were an integral part of programs that were associated with significant reductions in malnutrition (43). Further, it provides an opportunity to promote and encourage breastfeeding and appropriate complementary feeding which results in good nutrition status of children (43). In Nepal, apart from health facilities, growth monitoring sessions are conducted in at least three to five places within the community at the primary health care/primary outreach clinics (PHC/ORC) on a monthly basis. The national nutrition program has recommended that all children need to be growth-monitored at least six times in the first year, four times in the second year, and three times each in remaining three years (44). Such intervention increases the access to child health services to mothers who live in hard-to-reach areas from the health facilities. Growth monitoring is the entry point which determines children’s nutritional status and conducts counseling to the parents/caretakers for improved nutritional knowledge, attitude, and household level practices.

The findings from the current study have several implications for policy, research, and practice. Comprehensive interventions which focus on educating future mothers, improving MCH service utilization, and improving the economic status of households are required to improve the nutritional situation of children under 5 years of age. Improved feeding practices of pre-school children should be promoted. An earlier study suggested enhancing complementary feeding practice in Nepal by focusing on women from poorer households and those less exposed to media for the better nutritional status of children (29). Strategies to improve the nutritional status of children include improving women’s educational status (25) and promoting regular growth monitoring practices. These provide an opportunity for counseling mothers about their children’s nutritional needs and feeding practices. Furthermore, long-term strategies for improvement of economic status such as homestead food production, kitchen garden, and income-generating activities may also improve the child caring and feeding practices, which can impact nutritional status of children. Similarly, adequate health seeking practices during pregnancy may reduce the chance of low birth weight or preterm babies and if babies are born smaller than average size, more postnatal care, and extra care for such babies may minimize the possibility of being underweight in later months. Because of the high density of indigenous people living in the district (20), there might be many cultural and linguistic barriers to implementation of targeted interventions. More importantly, cultural practices such as eating less during pregnancy, poor food diversity need to be addressed by targeting pregnant women, families, and communities in behavior change communication activities (45). Mass media mobilization, distribution of IEC/BCC materials as per local context, counseling through peer mobilization (46), or involvement of mother-in-law in prenatal care (47) could be potential strategies to reduce such contextual barriers.

Some limitations of the present study should be acknowledged. A study conducted in eastern Nepal may not be generalizable to communities from other parts of the country, and the cross-sectional nature of the survey does not allow for inferences regarding causality. Self-reported response from the mothers is another limitation of this study, though probing questions were asked during interview and mothers had been invited to provide documents (for example birth certificate, antenatal care card, and others) to reduce the recall bias. Nonetheless, the strength of this study includes the objective assessment of the weight of the children and objective observations of some environmental factors during data collection.

## Conclusion

Underweight among under-five children was significantly associated with the age of a child, purified water consumption practices, growth monitoring practices, and the mother’s perception of small size at birth. These findings suggest the need for broader and longer-term interventions focusing on maternal and child nutrition promotion to reduce underweight among under-five children.

## Availability of Data and Material

The data and findings related to this research are included in the manuscript.

## Authors’ Information

DA holds an MPH and public health officer at the Ministry of Health Nepal. RK is a PhD candidate at The University Queensland School of Public Health, Australia. YP holds an MPH and is a Research Manager Nepal Health Sector Support Programme, Nepal. AP holds a PhD and is Associate Professor of Biostatistics at Tribhuvan University, Institute of Medicine, Department of Community Medicine and Public Health, Nepal.

## Author Contributions

DA designed the study concept and conducted statistical analysis of the manuscript. RK and YP contributed to data analysis, literature review, drafting and finalizing the manuscript. AP supervised the project. All the authors have read and approved the final version.

## Conflict of Interest Statement

The authors declare that the research was conducted in the absence of any commercial or financial relationships that could be construed as a potential conflict of interest.
